# Supplementary material for: Modelling Skylarks (Alauda arvensis) to Predict Impacts of Changes in Land Management and Policy: Development and Testing of an Agent-Based Model
Source: PLoS One. 2013 Jun 6;8(6):e65803. doi: 10.1371/journal.pone.0065803 (PMC3675089; doi:10.1371/journal.pone.0065803)
Supplement: Supporting Information S4 — The skylark ODdox as a zipped archive. (ZIP) [file pone.0065803.s004.zip › Skylark_ODdox/croprotation_8h.html]

ALMaSS Skylark ODdox: croprotation.h File Reference


ALMaSS Skylark ODdox
 2.0


- Main Page
- Related Pages
- Classes
- Files

- File List
- File Members

Classes |
Variables

croprotation.h File Reference

`#include <vector>`  
`#include "tov_declaration.h"`

|  |  |
| --- | --- |
| Classes | |
| class | CropRotation |
| struct | Rotation |
| struct | Starter |

|  |  |
| --- | --- |
| Variables | |
| class CropRotation \* | g\_rotation |
| const int | NoFarmTypes = 2 |

---

## Variable Documentation

class CropRotation\* g\_rotation

const int NoFarmTypes = 2

Referenced by CropRotation::CropRotation().


- CJT
- MSVC
- ALMaSS Working Source
- Landscape
- croprotation.h
- Generated on Thu Jan 10 2013 13:15:35 for ALMaSS Skylark ODdox by
   1.8.1.1
